# Therapeutic drug monitoring of corticosteroids/β_2_-agonists in the hair of patients with asthma: an open-label feasibility study

**DOI:** 10.3389/fphar.2023.1339835

**Published:** 2024-01-10

**Authors:** Hélène Salvator, Elodie Lamy, Camille Roquencourt, Emmanuelle Bardin, Philippe Devillier, Stanislas Grassin-Delyle

**Affiliations:** ^1^ Exhalomics^®^ , Hôpital Foch, Suresnes, France; ^2^ Service de Pneumologie, Hôpital Foch, Suresnes, France; ^3^ Laboratoire de Recherche en Pharmacologie Respiratoire—VIM Suresnes, UMR 0892, Université Paris-Saclay, Suresnes, France; ^4^ Université Paris-Saclay, UVSQ, INSERM, Infection et inflammation (2I), U1173, Département de Biotechnologie de La Santé, Montigny-le-Bretonneux, France; ^5^ Institut Necker Enfants Malades, U1151, Paris, France

**Keywords:** asthma, inhaled drugs, hair, therapeutic drug monitoring, budesonide, formoterol

## Abstract

**Background:** Although adherence to inhaled medication is critically important for treatment efficiency, around half of patients taking these drugs are non-adherent or make critical errors when using their delivery device. Segmental hair analysis might be a valuable tool for therapeutic monitoring because hair concentrations reflect exposure from month to month. The objective of the present proof-of-concept study was to establish the feasibility of segmental hair analysis of inhaled budesonide and formoterol in asthma patients.

**Methods:** We conducted a prospective, open-label, interventional study of adult patients being treated with budesonide/formoterol for controlled, moderate-to-severe asthma (CorticHair, NCT03691961). Asthma control, lung function, and medication adherence were recorded. Hair samples were taken 4 months after enrolment and cut into four 1 cm segments.

**Results:** Samples were available from 21 patients (20 women; median age: 53; median budesonide dose: 600 μg/d). Budesonide and formoterol were detected in samples from 18 to 13 patients, respectively. The median hair concentrations were 6.25 pg/mg for budesonide and 0.9 pg/mg for formoterol. The intrapatient coefficient of variation between hair segments was 21% for budesonide and 40% for formoterol. Pearson’s coefficients for the correlations between the hair concentration and the self-reported drug dose and the prescribed drug dose were respectively 0.42 (*p* = 0.08) and 0.29 (*p* = 0.25) for budesonide and 0.24 (*p* = 0.44) and 0.17 (*p* = 0.57) for formoterol.

**Conclusion:** Segmental hair analysis of inhaled medications was feasible, with low intrapatient variability. This innovative, non-invasive means of assessing monthly drug exposure might help physicians to personalize drug regimens for patients with difficult-to-treat asthma.

## 1 Introduction

Asthma is a chronic respiratory disease that affects 1%–18% of the population (GINA, 2022). The clinical manifestations and the treatment response vary markedly from one patient to another. Inhaled medications like corticosteroids (CSs) and long-acting β_2_-receptor agonists are the mainstay of asthma treatment. Patients with severe asthma are defined as those who require combination treatment with a high-dose inhaled or systemic corticosteroid and a controller medication (Ray et al., 2016). Although adherence is a key determinant of treatment efficacy, up to 75% of people with difficult-to-treat asthma are non-adherent (Cochrane et al., 2000; Gamble et al., 2009; GINA, 2022) and only 46%–59% of patients use their inhaler correctly (Cochrane et al., 2000). Poor adherence to inhaled medications and device misuse are associated with poor disease outcomes (Kocks et al., 2018) and can lead to unnecessary treatment escalation, such as the prescription of expensive add-on type 2 biologics that should be reserved for disease that is not controlled by high-maintenance-dose CS-based therapy.

Although therapeutic drug monitoring (TDM) of inhaled drugs can be an effective tool for optimizing treatment regimens and improving patient outcomes, new methods are needed. The conventional measurement of a plasma drug concentration only reflects short-term exposure and is therefore inappropriate for assessing long-term adherence. Hair analysis is a promising method for TDM because it allows the measurement of drug exposure over a longer period of time: drugs and their metabolites can become incorporated into the hair as it grows and thus provide a historical record of drug exposure. In this respect, segmental hair analysis allows an assessment of monthly drug exposure because hair grows by approximately 1 cm per month (Pecoraro et al., 1990; LeBeau et al., 2011). Although hair analysis has been used for TDM of several drugs [including antiretrovirals, antituberculosis drugs, antiepileptics and antipsychotics (Williams et al., 2001; Gandhi et al., 2011; van Zyl et al., 2011; Liu et al., 2014; Baxi et al., 2015; Gandhi et al., 2019; Metcalfe et al., 2019; Sun et al., 2019)], there are challenges in the application to asthma patients; the doses of inhaled drugs are low (with tens to hundreds of micrograms), as is the systemic bioavailability of the active compounds (Winkler et al., 2004). However, the incorporation of CSs (endogenous hormones, prednisone, methylprednisolone, triamcinolone, betamethasone, and beclomethasone) into human hair has already been documented in forensic, doping or clinical case reports (Cirimele et al., 1999; Bevalot et al., 2000; Cirimele et al., 2000; Gaillard et al., 2000; Kintz et al., 2000; Noppe et al., 2015). The only report in asthma patients featured the CS fluticasone propionate and the β_2_-adrenoreceptor agonists salbutamol, salmeterol, formoterol and vilanterol but did not assess monthly exposure in a segmental analysis (Hassall et al., 2018). The objective of the present proof-of-concept study was therefore to establish the feasibility of segmental hair analysis of inhaled budesonide and formoterol exposure in patients with asthma.

## 2 Methods

### 2.1 Study design and participants

We conducted a prospective, open, interventional study (“CorticHair”) in a university hospital (Foch Hospital, Suresnes, France). The study protocol was registered (NCT03691961) and approved by an independent ethics committee (CPP Sud Méditerranée III, 2018.02.05). Written, informed consent was obtained from all the participants. The participants were adult outpatients with asthma, aged between 18 and 65, having received a fixed-dose combination of budesonide/formoterol with a constant daily dose of budesonide ≥400 μg for more than 2 months, and with controlled or partly controlled asthma [according to the Global Initiative for Asthma (GINA) guidelines (GINA, 2022)]. The main non-inclusion criteria were pregnancy, any condition that could alter drug pharmacokinetics (known kidney or liver disease, comedication with other budesonide- or formoterol-containing drugs or CYP3A4 inducers/inhibitors), short hair (<5 cm), and hair implants or extensions. We planned to include 24 patients in the study.

### 2.2 Study measurements and procedures

All demographic and physical data were recorded at enrolment. The study period was 4 months and comprised a visit at inclusion and a visit at the end of the study. At each visit, lung function was measured with a standardized spirometry technique (Miller et al., 2005), the inhaler technique was checked, and asthma control was assessed with the GINA asthma symptom control questionnaire (GINA, 2022) and the Asthma Control Questionnaire (ACQ) (Juniper et al., 1999). Patients also filled out a daily medication adherence diary. Adherence was assessed as the ratio between the number of doses taken and the number of doses prescribed. Hair was sampled at the end-of-study visit; the sampling technique complied with the Society of Hair Testing guidelines (Cooper et al., 2012) and the European guidelines on workplace drug and alcohol testing in hair (Salomone et al., 2016). A strand of hair as wide as a thin pencil was cut from the vertex posterior region of the scalp with clean scissors and as close to the scalp as possible. The root end of the sample was clearly identified, secured with aluminium foil, placed in an envelope, and stored in a dry, dark environment at room temperature and away from direct sunlight (LeBeau et al., 2011; Salomone et al., 2016). The hair’s colour, length, structure and sampling site and any obvious cosmetic treatments were recorded. For the analysis, each hair sample was cut from the proximal end into four 1 cm segments. Budesonide and formoterol were measured using a liquid chromatography-mass spectrometry technique, as described previously (Lamy et al., 2020).

### 2.3 Data expression and statistical analysis

Measured drug concentrations were either expressed directly as pg/mg of hair or after normalization against the drug dose (pg/mg of hair/µg of drug). Missing values were not imputed. A Shapiro-Wilk test was conducted to check the assumption of normality. When normality was not established, a non-parametric test was performed. Pearson’s coefficient was calculated to assess the correlation between drug concentrations and continuous covariates of interest. For discontinuous covariates, a Kruskal–Wallis test was applied. The threshold for statistical significance was set to *p* < 0.05.

## 3 Results

### 3.1 The study population and treatment adherence

Twenty-four patients were enrolled between September 2018 and December 2021. Three patients were lost to follow-up, and so hair samples were available from 21 participants (Tables 1, 2). The median (interquartile range) self-reported adherence rate over the four-month study period was 100% [88%–100%].

**TABLE 1:** Patient characteristics and treatments.

sex (m/f)	1/20
Age (years)	52.7 [44.3–61.5]
Bodyweight (kg)	65 [59–74]
Body mass index (kg/m^2^)	24.0 [21.9–27.0]
Age at diagnosis (years)	41.0 [28.5–49.3]
Treatment duration (years)	3.0 [1.1–6.2]
Prescribed daily dose (µg budesonide/µg formoterol)	12
400/12	1
600/18	3
800/24	5
1600/48	
Hair colour (*n*)	4
Blond	3
Grey	11
Brown	2
Black	1
White	
Hair structure (*n*)	7
Straight	10
Wavy	2
Curly	2
Frizzy	

Continuous data are presented as the median (interquartile range).

**TABLE 2:** Lung function and asthma control.

	Visit 1	Visit 2
FEV_1_ (L)	2.5 [2.1–2.9]	2.5 [2.1–3.1]
FEV_1_ (%)	96 [88–105]	101 [83–106]
ACQ score	0.7 [0.5–1.1]	0.6 [0.2–1.4]
GINA (*n*):		
0	12	18
1–2	8	3
3–4	1	0

Continuous data are presented as the median (interquartile range). FEV_1_: forced expiratory volume in 1 min; ACQ: asthma control questionnaire; GINA: global initiative for asthma.

### 3.2 Hair analysis

#### 3.2.1 Hair drug concentrations

**FIGURE 1:**
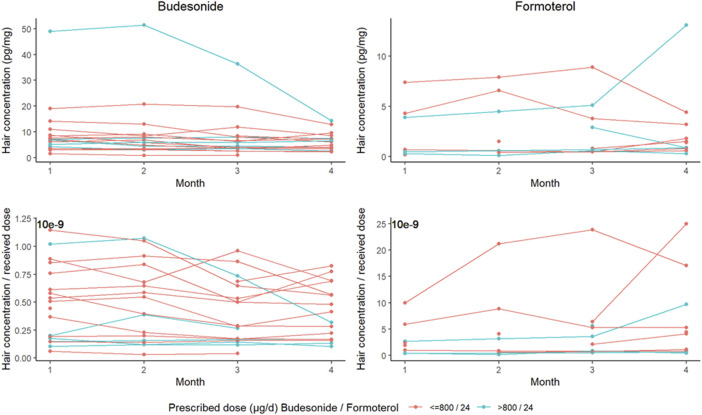
Hair concentrations of budesonide (left-hand graphs) and formoterol (right-hand graphs). The upper graphs show the unadjusted measured concentrations, and the lower graphs show the dose-normalized concentrations. Dose groups are shown in red (≤800 µg budesonide/day) and blue (>800 µg budesonide/day).

Budesonide was detected in 70 hair segments from 18 patients at a median (interquartile range) concentration of 6.25 [3.6–8.5] pg/mg (0.40 [0.17–0.68].10^−9^ pg/mg/µg when normalized against the dose received) (Figure 1). Formoterol was detected in only 37 segments from 13 patients at very low concentrations: 0.9 [0.6–3.9] pg/mg in absolute terms and 2.5 [0.7–5.7] pg/mg/µg when normalized against the prescribed dose.

#### 3.2.2 Intra- and inter-patient variability

Intrapatient variability was assessed in terms of the coefficient of variation over the 4-month study period, which was 20.8% [13.2–31.4] for budesonide and 39.9% [28.6–64.7] for formoterol. The influence of normalization against the received dose on interpatient variability was assessed through the coefficient of variation for the drug concentration in the first hair segment. For budesonide, this coefficient was 109% before normalization and 69.7% after normalization; for formoterol, the values were respectively 118% and 108%.

There were no between-month differences in the measured concentration of budesonide or formoterol, with or without dose normalization (*p–*values in an analysis of variance >0.663).

#### 3.2.3 The correlation between budesonide and formoterol hair concentrations

**FIGURE 2:**
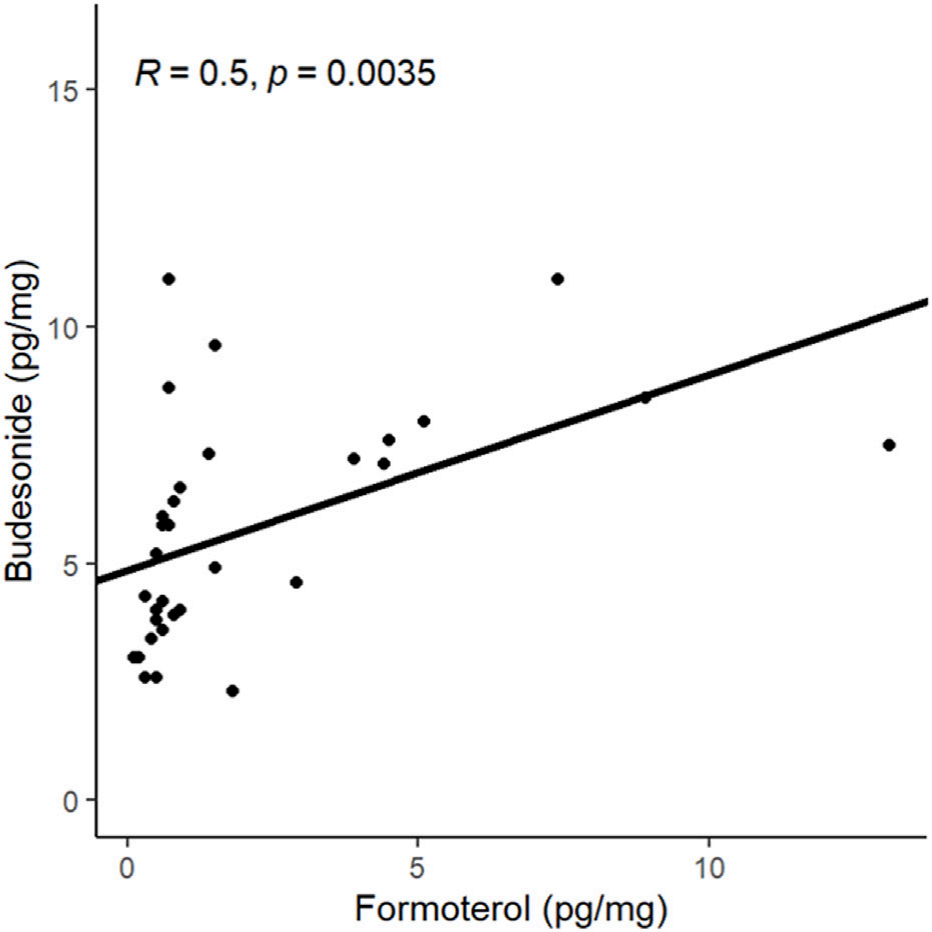
Analysis of the correlation between the hair concentrations of budesonide and formoterol.

Thirty-two samples contained both budesonide and formoterol. In a linear regression, Pearson’s coefficient for the correlation between the two drugs was 0.50 (*p* = 0.0035) (Figure 2).

### 3.3 Relationship between the hair drug concentration and inhaled medication use

**FIGURE 3:**
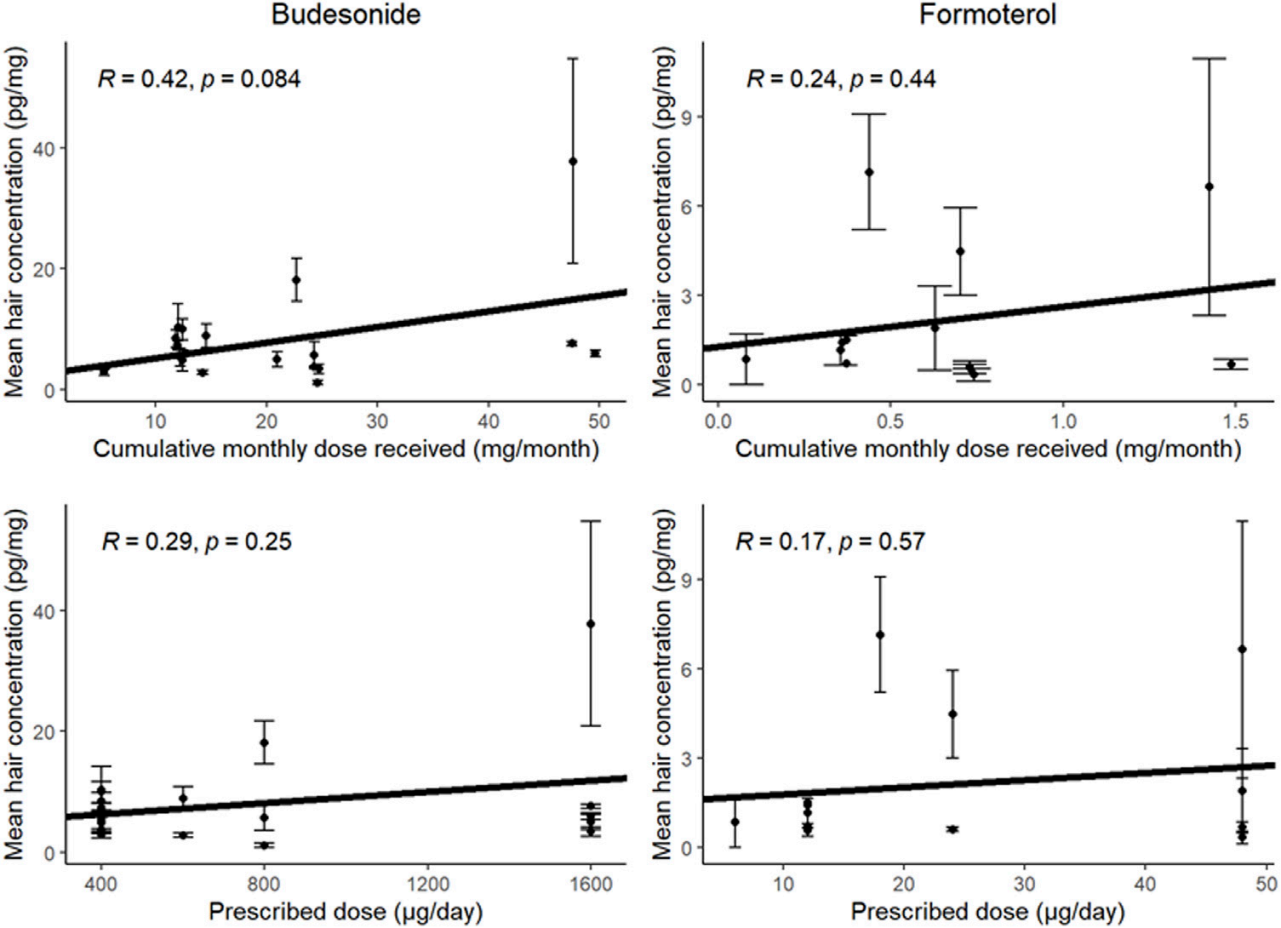
Analysis of the correlation between the mean hair concentrations of budesonide (left-hand graphs) or formoterol (right-hand graphs) and the cumulative monthly dose received (according to self-reports; top graphs) or prescribed (bottom graphs). Error bars represent standard deviation.

The mean hair concentrations for each drug over the 4 months were used to analyzed the correlations with the doses of inhaled medications. When the prescribed dose was considered, Pearson’s correlation coefficient was 0.29 (*p* = 0.25) for budesonide and 0.17 (*p* = 0.57) for formoterol (Figure 3). After the self-reported adherence rate was taken into account, the coefficient for the correlation with the cumulative monthly dose was 0.42 for budesonide (*p* = 0.08) and 0.24 for formoterol (*p* = 0.44).

### 3.4 Associations between hair drug concentration, patient demographics, lung function, and asthma control

**FIGURE 4:**
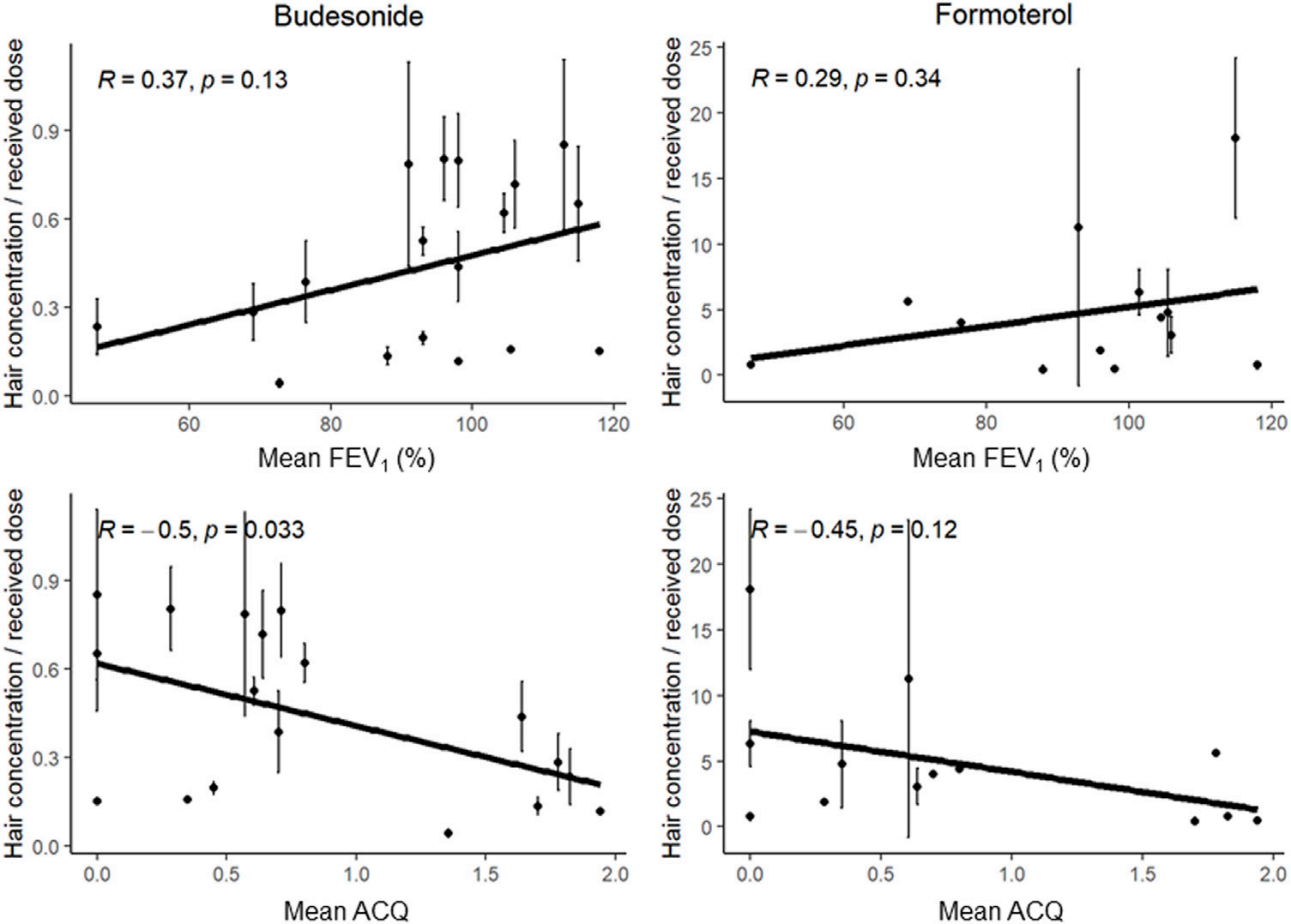
Analysis of the correlation between the dose-normalized hair concentrations of budesonide (left-hand graphs) or formoterol (right-hand graphs) and the mean FEV_1_ (top graphs) or the ACQ score (bottom graphs). Error bars represent standard deviation.

Firstly, we did not observe any significant correlation between the hair drug concentrations on one hand and patient demographics (age, bodyweight, height, and body mass index), hair colour and hair structure on the other. Secondly, we explored the association between hair concentrations on one hand and lung function and asthma control on the other. As the lung function and asthma control values at the inclusion visit and final visit were similar (Table 2), we considered mean values in the analysis. For the budesonide hair concentration, the correlation coefficient was 0.37 (*p* = 0.13) with FEV_1_ (%) and −0.50 (*p* = 0.033) with the ACQ score (Figure 4). For formoterol, the values were 0.29 (*p* = 0.34) with FEV_1_ and -0.45 (*p* = 0.12) with the ACQ score.

## 4 Discussion

Our study’s primary objective was met: we were able to quantify budesonide and formoterol in hair segments. Hence, segmental hair analysis might be a valuable tool for monitoring exposure to inhaled drugs in patients with difficult-to-treat asthma.

Budesonide and formoterol were detected in respectively 83% and 44% of the available hair samples. The difference in the proportion of samples with undetectable concentrations might be due (at least in part) to drug-related parameters (e.g., the intrinsic physicochemical properties that determine a drug’s incorporation into the hair), patient-related factors (as discussed below) and - above all - quantitative aspects. The inhaled daily doses were indeed very low, with tens to hundreds of micrograms. The airway deposition of drugs administered through the inhaled route is about 30%–35% of the administered dose, with the rest being retained in the mouth and oropharynx and eventually swallowed. Since budesonide has a very high first pass effect (up to 90% being metabolized to 6β-hydroxybudesonide and 16α-hydroxyprednisolone), budesonide hair concentrations are expected to be low. The dose of formoterol was 33-fold lower than that of budesonide. Hence, systemic exposure was low; the bioavailability of inhaled budesonide is about 18% only (Winkler et al., 2004), making the analytical method’s lower limit of quantification a critical parameter (Hassall et al., 2018). Furthermore, the concentrations in consecutive hair segments in patients receiving constant doses were remarkably stable; this was particularly true for budesonide, with an intra-patient coefficient of variation of 21%. This finding emphasizes the method’s potential value for assessing patient exposure over time. Expression of the detected concentrations as a function of the dose received was also helped to standardize the measurements and decrease inter-individual differences. Although the correlation coefficient between the hair budesonide concentration and the dose received was only 0.42 (not significant), our proof-of-concept study was not powered to reliably reveal this secondary outcome; a stronger exposure/concentration correlation might be apparent in a larger study. Our results are in line with those of a study in which the drug concentrations measured in a single hair sample were correlated with the level of adherence (Hassall et al., 2018). In this respect, the relationships between lung function, asthma control, and the hair drug concentrations must now be characterized in more depth. Here, we studied patients with controlled or partly controlled asthma, constant doses of medications, and similar lung parameters at the inclusion and final visits. Hence, we were unable to observe individual changes in lung function or analyze the latter’s relationship with hair drug concentrations. The same conclusions can be drawn from the association between the hair drug concentrations and the level of asthma control (as evaluated by the ACQ score); the data suggested a trend towards better asthma control with higher drug concentrations.

The present study had a number of strengths. Firstly, segmental hair analysis was used here for the first time with inhaled asthma medications. Blood and capillary concentrations do not reflect medication exposure in the same way. While blood concentrations represent the exposure of the last 24–48 h, capillary concentrations allow a retrospective assessment of drug exposure over several months, as the molecules present in the body at a given period are incorporated into hair and then remain during hair growth. The extent of drug incorporation reflects drug pharmacokinetics. For instance, after a single-dose exposure, a drug will be incorporated into the forming hair (proximal part) but not detected in the older hair (distal part). In contrast, in a patient undergoing regular daily treatment, drugs will be detected throughout the entire length of the hair strand. We used 1 cm segments, which reflects drug exposure over a period of 1 month; the mean ± standard deviation (range) growth rate of scalp hair is reportedly 1.06 ± 0.06 (0.6–2.2) cm/month (Pecoraro et al., 1990; Potsch, 1996; LeBeau et al., 2011). The principles of segmental hair analysis rest the underlying assumptions that substances and their byproducts integrate into the hair shaft during its growth, and the hair grows at an average rate of approximately 1 cm per month for each individual. By dividing the hair into 1 cm segments and analysing each segment, it becomes possible to estimate a one-month timeframe for detecting drug exposure (Pecoraro et al., 1990; Pragst and Balikova, 2006). Hair was also sampled from the vertex region of the scalp. This region is recommended for pharmacological investigations because it has the fastest growth rate and the highest proportion of follicles in the anagen (hair growth) phase (LeBeau et al., 2011). The study’s main limitations were related to its sample size. Our observations will require external validation in another cohort and must be extended to larger cohorts for the investigation of potential patient-related and other confounding factors, as well as to establish clinical applicability. Firstly, the skewed sex ratio prevented us from assessing the effect of sex; the short hair length non-inclusion criteria meant that few men participated in the study, and the hair growth rate is reportedly around 8% faster in females than in males (Van Neste and Rushton, 2016). The availability of hair of sufficient length is one of the limiting factors of the technique. Although alternatives exist, such as the analysis of hair from other regions (pubic, axillary, etc.), conducting segmental analysis on these hairs is not practically feasible. Moreover, interpretation becomes challenging due to increased variability in the growth rate of hair in these areas. The investigation of other potential confounding factors [such as ethnical origin (Saint Olivie Baque et al., 2012; Loussouarn et al., 2016), hair pigmentation (Van Neste, 2004; Choi et al., 2011) or comedications (Tosi et al., 1994)] was also outside the scope of the present study. It has been reported that some of these factors influence the hair growth rate. For example, anticancer therapies are well known to induce hair loss, while anabolic steroids, testosterone, ciclosporin or minoxidil may induce hair growth (Tosi et al., 1994). The factors’ possible impact on hair levels of inhaled medications remains to be characterized in detail. Indeed, the literature data suggest that formoterol concentrations may be higher in dark-haired individuals than in light-haired individuals (Hassall et al., 2018). However, all the hairs from a given individual would be similarly affected by hair treatments like bleaching and dyeing. These cosmetic treatments would not necessarily affect (semi)quantitative segmental hair analyses of between-month variations in drug exposure. Finally, there are known weaknesses in using patient self-reported observance for the data normalization, and the use of connected devices in future studies could offer certain advantages.

Taken as a whole, our results proved that segmental hair analysis has potential value for the therapeutic monitoring of inhaled medications in cases of difficult-to-treat asthma. The individual patients’ hair concentrations reflects the level of adherence and the appropriateness of the inhalation technique. The main purpose of the approach described here is the non-invasive evaluation of monthly drug exposure. Although the analysis is labour intensive, it is no more costly than other routine mass spectrometry analyses of drug levels. The results of a segmental hair analysis might help the clinician to personalise the drug regimen - especially when scaling-up to expensive biotherapies is being considered. Subsequent validation studies might help to determine the value of segmental hair analysis for the therapeutic monitoring of inhaled medications other than budesonide and formoterol and for patients with lung diseases other than severe asthma.

## Data Availability

The original contributions presented in the study are included in the article/supplementary materials, further inquiries can be directed to the corresponding authors.
